# Preparation and characterization of nanoclay-hydrogel composite support-bath for bioprinting of complex structures

**DOI:** 10.1038/s41598-020-61606-x

**Published:** 2020-03-24

**Authors:** Ferdows Afghah, Mine Altunbek, Caner Dikyol, Bahattin Koc

**Affiliations:** 1 Sabanci Nanotechnology Research and Application Center, Istanbul, 34956 Turkey; 2 Sabanci University Faculty of Engineering and Natural Sciences, Istanbul, 34956 Turkey; 3 Sabanci University Integrated Manufacturing Technologies Research and Application Center, Istanbul, 34906 Turkey

**Keywords:** Biomaterials, Tissue engineering

## Abstract

Three-dimensional bioprinting of cell-laden hydrogels in a sacrificial support-bath has recently emerged as a potential solution for fabricating complex biological structures. Physical properties of the support-bath strongly influence the bioprinting process and the outcome of the fabricated constructs. In this study, we reported the application of a composite Pluronic-nanoclay support-bath including calcium ions as the crosslinking agent for bioprinting of cell-laden alginate-based hydrogels. By tuning the rheological properties, a shear-thinning composite support-bath with fast self-recovery behavior was yielded, which allowed continuous printing of complex and large-scale structures. The printed structures were easily and efficiently harvested from the support-bath without disturbing their shape fidelity. Moreover, the results showed that support-bath assisted bioprinting process did not influence the viability of cells encapsulated within hydrogel. This study demonstrates that Pluronic-nanoclay support-bath can be utilized for bioprinting of complex, cell-laden constructs for vascular and other tissue engineering applications.

## Introduction

Three-dimensional (3D) bioprinting provides controlled deposition of hydrogels, biological matters or biomaterials to fabricate complex cell-laden structures in a layer-by-layer manner for various tissue engineering applications. Natural or synthetic biocompatible and biodegradable cell-laden hydrogels are commonly used to construct 3D environment with the ability to turn into gel at physiological conditions without impairing cell integrity and cell-to-cell interaction. Extrusion based bioprinting is one of the most common method to deposit cell-laden hydrogels in a desired geometry with precise control in micrometer scale. The process requires gelation of liquid hydrogel either by physical, thermal or chemical crosslinking before, during, or after bioprinting. However, physical phase transition of hydrogels during extrusion might clog the nozzle and could disrupt the viability of the encapsulated cells^1,2^. In addition, due to low mechanical strength, the printed hydrogels may not be strong enough to hold overhanging structures. Integration of the subsequent layers is another challenge which needs proper adjustment of hydrogel gelation time with the printing process^3,4^. The level of humidity strongly affects cellular viability, which is not often preserved during in-air hydrogel extrusion bioprinting^5,6^. These limitations can arise due to both hydrogel properties such as viscosity and gelation time, and the printing parameters such as fabrication time, extrusion pressure and nozzle size. Among them, viscosity of the hydrogel has a pivotal role. Viscosity can be fine-tuned with increasing the concentration, which increases the hydrogel stiffness and subsequently might have an adverse effect on cell migration and functioning. A sacrificial secondary hydrogel with different gelation property, or a viscosity modifying biomaterial is generally introduced within the primary hydrogel to obtain a qualified structure during the extrusion based bioprinting process^7–10^.

Direct free form writing of hydrogels in a fugitive and sacrificial support-bath has addressed aforementioned limitations. A support-bath with the Bingham plastic flow behavior can provide a rigid supporting matrix and at the same time, instantaneous yielding and rapid recovery during and after passage of the extruding nozzle, respectively^6,11–14^. In addition to the adequate flow behavior, the support-bath should quickly provide the necessary gelation to control the spreading of the extruded viscous bioink and let the printed layers to be integrated, and concurrently avoiding nozzle clogging. This approach has been demonstrated by depositing liquid hydrogel precursors within self-healing support materials such as Carbopol, Laponite, gelatin, gellan, fumed silica particles, Pluronic and alginate^15–22^. However, the functionality of the support-bath materials is influenced by several parameters. In addition, the compatibility of the support-bath with the printed hydrogel has also a crucial role for a successful bioprinting^15–18,23^. For example, hydrophobic perfluorotributylamine fluid was employed for the bioprinting of agarose hydrogel due to its high buoyant density^17^. Since the approach of supporting is based on buoyancy, viscosity of the printed hydrogel might affect the structural resolution which limits the applicability of this support material in different types of hydrogels. In another study, two different types of hyaluronic acid which were modified with adamantane or β- cyclodextrin, respectively, were utilized as a self-healing support material, by using their reverse assembly capability as host-guest interactions ^23^. Although methacrylated gels were successfully printed, the possible reaction of adamantane or β-cyclodextrin ends would limit the utilization of this technique to be used with different materials. Due to their stress-yielding properties, Carbopol microgels and gelatin microparticles have also been studied^15,16^. However, ionic sensitivity of the Carbopol, and thermal instability and microparticle size-dependency of the gelatin slurry limit their use. Therefore, to address limitations and general requirements for bioprinting of hydrogels with various properties, new formulations of support-bath systems are needed.

Poly(ethylene oxide)-poly(propylene oxide)-poly(ethylene oxide) (Pluronic F127; PF) is one of the support-bath material candidates possessing concentration dependent-thermoreversible gelation property. It is in gel form at around body temperature (concentrations > 18%) and turns into liquid below 10 °C^24^. Hence, it was implemented as support-bath or sacrificial fugitive ink at room temperature within the range of 25–40% concentrations^25,26^. However, viscoelastic modulus of the material was not strong enough for micrometer scale resolution in a long time printing processes due to mechanical weakness and tendency of quick dissolving in physiological conditions^24,26^. Sol-gel transition concentration of PF was modified by addition of Laponite^8,27^. Laponite is a layered synthetic nanoclay with chemical formula of Si_8_Mg_5.45_Li_0.4_O_24_Na_0.7_ similar to hectorite. It exists as a 2D disc-like structure, 25 nm in diameter and 1 nm in thickness with negative charges distributed on the faces (OH^−^) and positive charges on the edges (Na^+^). Due to its biocompatibility, low cost, availability, thermal stability, processability, ionic insensitivity, and anisotropic behavior, Laponite can be considered as a promising rheology-modifier, or used as mechanical reinforcing component and crosslinker with several hydrogel systems^28–30^. It was utilized in different applications of tissue engineering from composite hydrogel printing to support-bath material^14,31–34^. The gel-forming ability of Laponite involves a multi-step mechanism. When particles react with hydroxide ions in the water, phosphate ions dissolve. After the ion dissolution, the nanoclay particles start to interact with each other while the sodium ions diffuse towards the surfaces within the galleries, resulting in expanded thixotropic gel structure^35–37^. Laponite RDS, a category of Laponite family, possesses an extra peptizing agent of sodium pyrophosphate (Na_4_P_2_O_7_) at the edges which ionically stabilizes the structures and prevent the face-edge bond formation between particles^38^. The pyrophosphates give a thixotropic behavior to the structure^38^. These properties of Laponite would make it a suitable support-bath material, but the printed hydrogels have high viscosity with stiff network which is a disadvantage for cellular activities like cell adhesion, migration and proliferation^39–41^. In addition, the removal procedure of the supporting gel is complicated, often resulting in damage to the printed structure due to being strongly adherent nanoclay particles^22^.

In this study, we developed a composite support-bath based on the mixture of PF and Laponite (PF-RDS) in the presence of calcium ions, to be used in free form bioprinting of complex cell-laden hydrogel structures. Although both materials show unique properties and have been individually employed as sacrificial support gels, they showed limited capacities in bioprinting of low viscosity inks at low concentrations and also the ease and efficiency of removal procedure^4,14^. By combining two components as a composite support-bath, it would be possible to employ distinct characteristics of each, namely the thermoresponsive gelation of PF and the thixotropic behavior of Laponite. We analyzed different formulations with varying concentrations of PF-RDS and calcium chloride (CaCl_2_) to achieve optimum rheological properties. Sodium alginate was utilized as a precursor solution to evaluate printability of complex and hollow structures by *in situ* crosslinking within the support-bath. Finally, cell-laden hydrogel structures were bioprinted in the support-bath and the cytocompability of the bioprinting process was analyzed by monitoring the viability after printing process.

## Results and Discussion

### Rheological characterization of the support-bath

The weak interactions of the support-bath components play a critical role in the formation of network and its stability and hence the preservation of the printed hydrogel structures in over-hanged and complex geometries^11–13^. In this study, thermoresponsive behavior of PF was combined with the thixotropic behavior of the Laponite to obtain yielding and easily removable support-bath structure. Laponite RDS was selected due to formation of a stable polymer-nanoclay complex with the presence of Ca^2+^ and possession of a neutral isoelectric point providing a biocompatible environment^37,38^.

**Figure 1:**
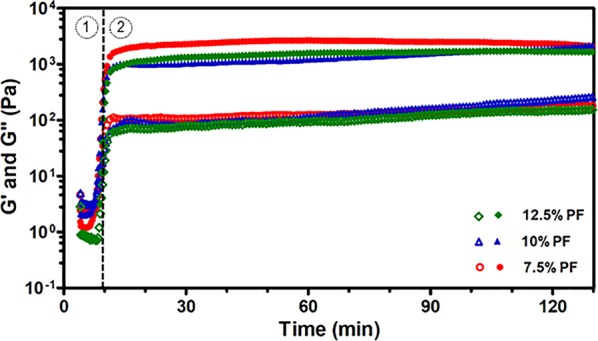
Temperature and time sweep measurements of the support-bath showing storage and loss moduli over time for different concentrations of PF. Laponite and CaCl_2_ concentrations were set to 3% and 1%, respectively. Storage (G′) modulus (filled symbols) and loss (G″) modulus (open symbols). Vertical dashed line seperates two measurements. Region (1) represents temperature sweep test from 4 to 37 °C and region (2) shows time sweep measurement at 37 °C for 2 h.

Thermoresponsive gelation behavior of the PF-RDS support-bath with different formulations was characterized through a typical temperature sweep test from 4 to 37 °C with a final dwelling time of 2 h by the evolution of storage (G′) and loss (G″) moduli (Fig. 1). By increasing temperature, G′ and G″ moduli raised as a result of both liquid crystalline phase transition of PF in ionic media and the presence of Laponite particles^9,27,37^. Then, G′ and G″ reached a steady state by further incubation at 37 °C.

### Effect of PF concentration

**Figure 2:**
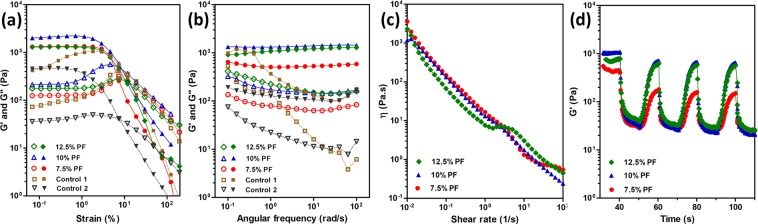
Dynamic rheological characterization of the support-bath representing the effect of PF concentration on flow behavior and recoverability of the structure at 37 °C (Laponite-RDS and CaCl_2_ concentrations were constant at 3 and 1%, respectively except for control samples). Control 1 and control 2 included 10% PF, and 3% RDS, respectively at constant 1% CaCl_2_ (**a**) Strain amplitude sweep profiles of supporting mediums, (**b**) frequency sweep profiles within the linear viscoelastic range, (**c**) viscosity vs. shear rate plots revealing the shear thinning behavior of the support material, (**d**) cyclic strain measurements at high (50%) and low (0.6%) strains showing storage (G′) moduli of the samples in 4 cycles. Storage (G′) modulus (filled symbols) and loss (G″) modulus (open symbols).

Figure 2 represents rheological characterization of different concentrations of PF in the presence of Laponite-RDS and CaCl_2_ at constant concentration. Dynamic moduli of composites containing 7.5, 10, and 12.5% of PF, and 3% Laponite RDS, and 1% CaCl_2_ are plotted during the strain amplitude (Fig. 2a) and angular frequency sweeps (Fig. 2b). Strain amplitude graphs demonstrate the flow point of the materials at which a transition between elastic gel state (G′ > G″) to viscous liquid-like state (G″ > G′) is observed^34,42^. At very low strain amplitudes, G′ and G″ values show the linear viscoelastic behavior^43^. Increasing the concentration of PF from 7.5% to 10% in the composite resulted in an apparent increase in both moduli during constant strain amplitude loading, however further increase to 12.5% induced a significant decrease (Fig. 2a). The value of storage moduli of 7.5 and 12.5% PF containing composites were observed to be almost the same while the corresponding value for 10% PF one was 1.5-fold higher. The interactions of PF and RDS are considered to be very complex and have not been completely known, however it is assumed that electrostatic interactions as well as hydrogen bonding are the dominant players in the final structural configuration^33,44^. Reversible adsorption and detachment of polymer chains on the charged surfaces of the Laponite nanodiscs would result in a physically crosslinked network which shows solid-like elastic or fluid-like viscous behavior depending on external forces. Furthermore, cluster formation of the clay nanodiscs and subsequent agglomeration with PF polymeric chains would contribute in observation of high yield behavior upon applied stress^42,45^. The density of charges at the edges of Laponite RDS is different associated with pyrophosphate ions, which will lead to face-face interaction in contrast to face-edge attraction^35,46^. The attraction between these bonds will further link more particles at the face-edge orientation and create a network that forms the final “gel state”^42,47,48^. The observed developments in both G′ and G″ by increasing the PF concentration can be attributed to the bridging effect of PF micelles and chains with their vicinal nanoclay particles which results in stabilization of the composite^33,49^. In this way, it is speculated that there would be a threshold for such interactions, which could be translated to the surface capacity of the Laponite-RDS in such a system. A viscoelastic gel structure could be formed by increasing the PF concentration up to a certain value above which, the faces of the Laponite-RDS nanoclays would be already saturated by the adsorbed polymer. Further increase in the polymer concentration will not necessarily contribute in establishment of long-range elastic interactions between the two components of the system^27,50^. The mixtures of PF and CaCl_2_ (Control 1) and Laponite-RDS and CaCl_2_ (Control 2) were selected as control groups to identify the contribution of each individual component of the composite in development of the viscoelastic properties. It should be noted that the exclusion of CaCl_2_ from the mixture of PF-Laponite RDS resulted in formation liquid-like composite, failing to form a viscoelastic gel network^37,38^. Hence, this composition was not included in the set of experiments. Control 1 failed to show a detectable linear viscoelastic limit within the range of strain values 10^−3^ to 10^3^ (%). This can be explained by formation of clusters consisting of polymer chains at low concentration of PF as reported elsewhere^51^. Control 2 showed a linear viscoelastic range during strain amplitude sweeps, however the storage modulus values were much lower than the corresponding region in PF-RDS composites containing PF at the same CaCl_2_ concentration^48^. Both control groups showed less elastic moduli, since Laponite-RDS acts as a physical cross-linker to PF polymer chains in the presence of CaCl_2_ which is in agreement with previous study of Wu *et al*.^8^.

The dynamic viscoelastic properties of the formed networks of PF in the presence of Laponite-RDS and CaCl_2_ were also probed by frequency sweep analysis (Fig. 2b). The elastic features of the matrix were dominant throughout the whole measured frequencies, characterized by G′ values higher than G″ values^42^. The elastic modulus value at 7.5% PF was considerably lower than the other two concentrations. By increasing the PF concentration to 10%, the interactions between PF and Laponite-RDS evolves from a viscoelastic-dominant gel state to a glassy state colloidal network, in which the elastic modulus is almost independent from the frequency of deformation. Further increase in PF concentration to 12.5% resulted in weakening of the elastic response of the system at low frequencies, an indication of the increased contribution of excess PF chains which are speculated to have no direct interactions with Laponite nanoparticles. The results demonstrate that concentration dependent interactions between polymer and clay nanoparticles allow the formation of suitable and stable network for support-bath^48,49,52^. Control 1 showed a strong dependency of both elastic and loss moduli to frequency values. It could be due to testing parameters which was not in a viscoelastic region. Control 2 showed glassy gel-like behavior with almost constant elastic modulus values at all frequencies, revealing the effect of calcium ions in the formation of House of Cards-like structure which was utilized as support-bath material in previous studies^14^. However, the value of storage modulus was much lower compared to the composite with PF which needs more viscous bioink to be able to provide enough mechanical strength to hold the structure during bioprinting process.

Shear thinning behavior and yield stress values of the composite support-bath materials were investigated by a shear rate sweep test (Fig. 2c). As the graph implies, all the tested concentrations showed the same trend of viscosity drop but the sample with 7.5% PF had the highest decrease in its viscosity while the sample with 10% of PF had the lowest change. The Herschel-Bulkley model (Eq. 1) was used to fit the data and estimate the dynamic yield stress and flow index values^15,35^.

1 $$\tau ={\tau }_{y}+K{\gamma }^{n}$$

where 𝜏 is shear stress, 𝜏_y_ is yield stress, 𝛾 is shear rate, and K and n are consistency factor and flow index, respectively. The model’s parameters are presented in Table 1.

**Table 1:** Herschel-Bulkley parameters for composite materials with different PF concentrations at constant concentrations of Laponite-RDS (3%), and CaCl_2_ (1%).

PF concentration (%)	𝜏_y_ (Pa)	K	n
7.5	32.8 ± 3.5	6.78 ± 2.2	0.73 ± 0.1
10	42.6 ± 5.7	10.32 ± 1.3	0.47 ± 0.2
12.5	28.6 ± 4.3	5.4 ± 2	0.60 ± 0.17

Flow index (n) is the main indicator of the shear-thinning behavior. The indices of 7.5 and 12.5% PF were almost equal, showing the same behavior in viscosity drop, which can be also observed in Fig. 2c. Yield stress values obtained from Herschel-Bulkley model are in agreement with the yield stress trends from strain sweep measurement. On the other hand, PF samples with 10% concentration showed an increase in yield stress, which could be attributed to the same saturation of polymer-particle interactions as mentioned previously. It should be noted that Control 2 samples did not show that much different values of viscosity vs. shear rate and Control 1 data was not reliable as mentioned in the previous part due to its non-linear viscoelasticity, therefore the graphs were not included.

Figure 2d shows recoverability of the composite throughout cyclic deformation. Due to the thixotropic characteristics, the disturbed matrix result in rebuilding of the interactions by forming the matrix network over time^4,34^. The so-called self-recovery property represents an essential feature of the composite matrix to be utilized as a support-bath material. Dynamic strain tests were performed at high (50%) and low (0.6%) strains. The strain cycles were repeated in 10 seconds intervals to monitor how fast the composite material could recover itself. Thixotropic behavior was monitored within 16 cycles (data was clipped to 4 cycles to enhance the legibility) and the recovery time and the extent of drop in storage moduli after 3^rd^ cycle of deformation were almost constant. As shown in the graph, even in a short time of 10 seconds the structures with 10 and 12.5% of PF could almost reach to their initial storage moduli. The composite with 7.5% of PF showed lower recovery in storage modulus compared with the starting point. This could be due to lower amount of polymer-chains which could not enhance the composite matrix stiffness^49^ as the amount of PF was not enough to resist the high shear strain values to rapidly recover the physically crosslinked polymer chains attaching on the Laponite nanodiscs charged surfaces^42,45^.

### Effect of CaCl_2_ concentration

**Figure 3:**
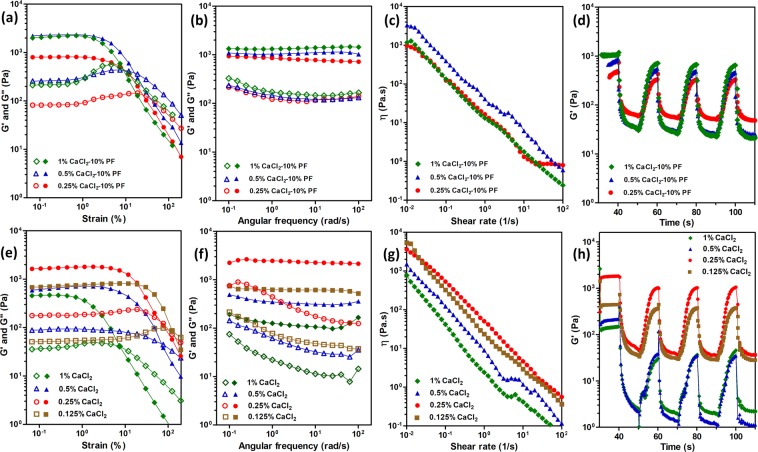
Dynamic rheological characterization of the support-bath representing the effect of CaCl_2_ concentration on network characteristics, flow behavior and recoverability at 37 °C (a-d were included samples with constant PF and Laponite concentrations of 10 and 3%, respectively.) (**e**–**f** were included only 3% Laponite-RDS.) (**a**,**e**) Strain amplitude sweep profiles of supporting medium, (**b**,**f**) frequency sweep profiles within the linear viscoelastic range. (**c**,**g**) viscosity vs. shear rate plots revealing the shear thinning behavior of the support material, (**d**,**h**) cyclic strain measurements at high (50%) and low (0.6%) strains showing storage (G′) moduli of the samples in four cycles. Storage (G′) modulus (filled symbols) and loss (G″) modulus (open symbols).

The contribution of ionic content in the formulation to viscoelastic properties of the composite was systematically assessed by varying the CaCl_2_ concentration with constant PF and Laponite-RDS which were set to 10 and 3%, respectively. We evaluated three different concentrations of 1, 0.5, and 0.25% for CaCl_2_ in the formulation to provide a moderate crosslinking for dispensed liquid form of alginate and to obtain an integrated structure without diffusion into the support-bath^35,48,53^. It should be mentioned that the lowest concentration of Ca^2+^ were above the threshold of “gel” formation below which a “glass-colloid” would be formed as explained in the previous study^54^. Figure 3 shows the rheological properties of the composites with different CaCl_2_ concentrations.

Strain amplitude sweep (Fig. 3a) shows the correlation of the extents of the linear viscoelastic region with three different CaCl_2_ concentrations. At low strain values, storage moduli are higher than loss moduli demonstrating the solid-like elastic behavior of the structures^43^. By further increasing strain values, structure starts to breakdown and material starts to yield. Dynamic moduli of the sample containing 0.25% CaCl_2_ was observed much lower than the other two concentrations since low ionic concentrations cannot lead to complete formation of aggregates^45,48^. Concentration of ions will change the net charge distribution of nanoclay particles in the composite matrix from edge to the surface and through the polymer chains^45^. The variation of surface charge in the composite gels with lowest CaCl_2_ content translates to the enhancement of Van der Waals forces compared to the ionic interactions. By increasing the salt content, the network formation will face a transition from repulsive clay-clay interactions to attraction of opposite charges and eventually forming a strong gel network^45^. The electrostatic attractive forces among nanoclay particles would correspond to material’s yield stress^46^. In addition, enhanced exfoliation of nanoclays with the increased ion concentration will take place that might correspond to the phase separation of the matrix under stress^48,55^. It was evident by evaluation of the obtained rheological data that increasing the ion concentration resulted in a similar trend in increasing the storage modulus. Considering these data, it is concluded that the ionic content in the current experimental design was still below the threshold to induce any phase separation^37,47,48^. It should be also noted that presence of divalent Ca^2+^ ions would shift the equilibrium in balancing of the ionic interactions between nanoclay galleries, which the acclaimed House of Cards-like structure during network formation of pristine Laponite is due to exfoliation of nanodiscs in presence of monovalent Na^+^ ions. In this way, formation of a long-ranged network due to exfoliation and collapse of nanoclay galleries in the absence of PF showed a threshold concentration of CaCl_2_ above which, the dynamic moduli and cross-over points in strain sweeps decreased significantly. We speculated that at this critical concentration of divalent Ca^2+^ ions, the exfoliation of nanodiscs was to an extent that the formed network was completely dependent on ionic interactions and secondary forces like Van der Waals interactions between PF and Laponite nanodiscs could not significantly contribute in overall viscoelasticity of the composite.

All the formulations showed a glassy state with frequency independence of elastic moduli throughout a wide range (Fig. 3b). The one order of magnitude ratio between G′ than G″ indicated a stable network formation with no relaxation time within the probed range of frequencies^12^. The sample with 0.25% CaCl_2_ showed more dependency of storage modulus to the frequency of deformation which is an indication of weaker and more viscous nanoclay-nanoclay and polymer-nanoclay interactions. It is speculated to be due to the contribution of Ca^2+^ in formation of the glassy House of Cards-like structure in PF-RDS^48^.

Shear-thinning behavior of the samples with different ionic concentrations was explored through a shear rate sweep measurement (Fig. 3c). Dynamic yield stress and flow index values were calculated by fitting the data to Herschel-Bulkley model (Table 2). For all the samples viscosity decreases by increasing shear rate expressing similar shear-thinning behavior regardless of their ionic concentration. However, the sample with 0.5% CaCl_2_ showed higher values which could be due to bridging effect of Ca^2+^ that is the connection of two nanoclay particles from their negative surfaces that results in agglomeration of particles and possessing higher viscosity^48^.

**Table 2:** Herschel-Bulkley parameters for composite materials with different CaCl_2_ concentrations at constant concentrations of Laponite-RDS (3%), and PF (10%).

CaCl_2_ concentration (%)	𝜏_y_ (Pa)	K	n
0.25	26.3 ± 5	7.3 ± 3.6	0.58 ± 0.1
0.5	47.7 ± 4.2	11.39 ± 3	0.27 ± 0.15
1	42.6 ± 5.7	10.32 ± 2.3	0.47 ± 0.2

Cyclic strain measurements were performed and storage modulus vs. time was plotted to explore the recoverability of the structures for 10 seconds per cycle. (Fig. 3d). Storage values dropped after first cycle and continued with almost the same values. The lowest CaCl_2_ concentration showed less decrease and less recovery in storage modulus during the test. This phenomenon could be attributed to the fact that the ionic concentration was not enough to interact with nanoclay particles and hence not much gel-like structure was formed^14^. Initial storage modulus values were higher for samples with higher ionic concentration which is in agreement with aforementioned structural properties.

Based on previously published article which utilized a composite of CaCl_2_-Laponite as a support-bath material^4^, we selected four different formulations of CaCl_2_ with 3% Laponite-RDS and explored how ionic concentrations would affect their rheological behavior. Figure 3e represents flow behavior of the samples at different strain values. Interestingly, by increasing CaCl_2_ concentration from 0.125 to 0.5%, G’ increased and afterwards decreased and the formulation of 0.25% CaCl_2_ showed higher storage modulus compared to other samples. Based on visual observation, composites containing 0.125 and 0.5% concentrations of CaCl_2_ formed stiffer gel-like structure than others. In addition, their flow points are at higher strain values which could be attributed to bridging effect^48^. Compared to composites containing PF, the agglomeration of nanoclay particles due to interaction of two negatively-charged surfaces happened at lower ionic concentrations. In other words, composites containing PF with 0.5% of CaCl_2_ showed more viscous properties, whereas samples correspond to 0.25% of CaCl_2_ without PF had higher viscosity and elastic moduli. This result demonstrates the effect of Ca^2+^ ions on Laponite nanoclay structure as shown in previous studies^14,19,48^.

Almost the same behavior was observed for frequency sweep measurements (Fig. 3f). The composites of CaCl_2_ and Laponite-RDS act as a glassy colloid with no dependency of elastic moduli on frequency changes. Elastic moduli values repeated the same as for strain amplitude sweep test. The more the ionic concentration, the more possibility to shield the nanoclay charges and consequently damaging the House of Cards-like structure of Laponite^56^.

Shear thinning behavior of the samples were monitored through a shear-rate measurement and their viscosity were plotted against shear rate values (Fig. 3g). As the graph implies, all the formulations represented shear-thinning behavior and their viscosity dropped by increasing shear rate values.

Recoverability of the samples were investigated through a cyclic strain measurements (each cycle was 10 sec) and corresponding storage modulus values were recorded and plotted vs. time to mimic the nozzle translation in the support-bath. The composites containing 0.125 and 0.25% of CaCl_2_ showed much higher G’ values with almost constant values for each cycle revealing their ability to recover their structure rapidly. The other two formulations containing 0.5 and 1% of CaCl_2_ had low storage moduli and their values drop too much after first cycle demonstrating that the composites are not capable of fast recovery. Compared to composites containing PF their storage moduli are extremely lower which is due to interaction of polymer molecular chains and nanoclay particles causing physical cross-linking^48^. This demonstrates that both thixotropic behavior of Laponite and thermoresponsive properties of PF contribute for the physical crosslinking and the formation of a viscoelastic support-bath.

It is noteworthy to mention that low concentrations of CaCl_2_ showed promising behavior to be used as an electrolyte together with Laponite nanoclay. In this study, we selected 0.5% CaCl_2_ since it was not only a structural modifier for Laponite, but also a cross- linker for alginate. Our aim was to utilize a low concentration cell-laden hydrogel (3% alginate) to provide a cell-friendly bioprinting process with the utilization of a relatively low pressure for extrusion of bioink in a moderate CaCl_2_ concentration to obtain sufficient cross-linking density for structural integrity among the subsequently printed layers.

### Printability of overhanging and complex structures in PF-RDS support-bath

The challenges of overhanging and tubular complex structure printing have been addressed with different approaches in the literature^57–62^. The principle of the printing mechanism of those tubular structures are based on one-step extrusion of hydrogel in air, which is cross-linked by the inclusion of crosslinker through a coaxial nozzle system. These techniques are capable of printing hollow vascular-like structures in small dimensions by employing different formulations of bioinks, whereas construction of complex shapes in a layer-by-layer manner may not be applicable for integration of the extruded subsequent layers.

Printing in support-bath has been an emerging approach utilized by extrusion of hydrogels in a liquid form to have an appropriate shape fidelity, as well as to provide a more cell-friendly process compared to in-air extrusion bioprinting^61^. Laponite support-bath was demonstrated as support bath for fabrication of overhanging and complex branched tubular structures for various hydrogel types with different crosslinking mechanism^14,63^. Although Laponite support bath was investigated individually with many aspects for printing, the extruded hydrogel concentration was very high which could affect living cell functionality^14^. The use of high concentration of hydrogel is expected due to the slow crosslinking kinetics of alginate in the presence of low concentration of CaCl_2_, which has a strong negative effect on viscoelastic properties of Laponite as it is demonstrated above.

Rheological characterization suggested that all the tested PF-RDS composites could be used as a support-bath for hydrogel printing. Three percent of alginate was used for printing inside the support-bath. Among PF concentrations, 10% showed better viscoelastic behavior in terms of storage modulus and self-recovery. Printability of alginate into the support-bath containing various CaCl_2_ concentrations were investigated. In the previous report, concentration of CaCl_2_ for the Laponite support-bath was 0.125%^4^. However, our initial experiments showed that even the CaCl_2_ concentration of 0.25% was not enough to efficiently cross-link 3% of alginate hydrogel during printing. This concentration was much lower than the hydrogel that was used in the mentioned study (8% alginate)^4,14,64^. On the other hand, increasing the concentration of CaCl_2_ to 1% resulted in fabrication of structures with non-integrated fibers and occurrence of staircase effect^14^. Hence, the support-bath with composition of 10% PF and 0.5% CaCl_2_ was used. Moreover, Ding *et al*. increased CaCl_2_ concentration to 0.5% in a 4% Laponite support bath, while they used a concentrated and more viscous hydrogel blend as the extruded bioink (3% alginate+10% gelatin)^14^. The use of highly viscous hydrogels could be correlated to the decreased self-recovery properties of Laponite support-bath in the presence of high CaCl_2_ concentrations. It is worth mentioning that increasing the concentration of CaCl_2_ above 0.125% causes the deterioration of the House of Cards-like structure and shear-thinning property of only Laponite support-bath. Formulation of the Laponite support bath with PF resulted in increased tolerance over the CaCl_2_ content and allowed to dispense less viscous hydrogel with higher shape fidelity at low extrusion pressure, which would be a more cell friendly process.

**Figure 4:**
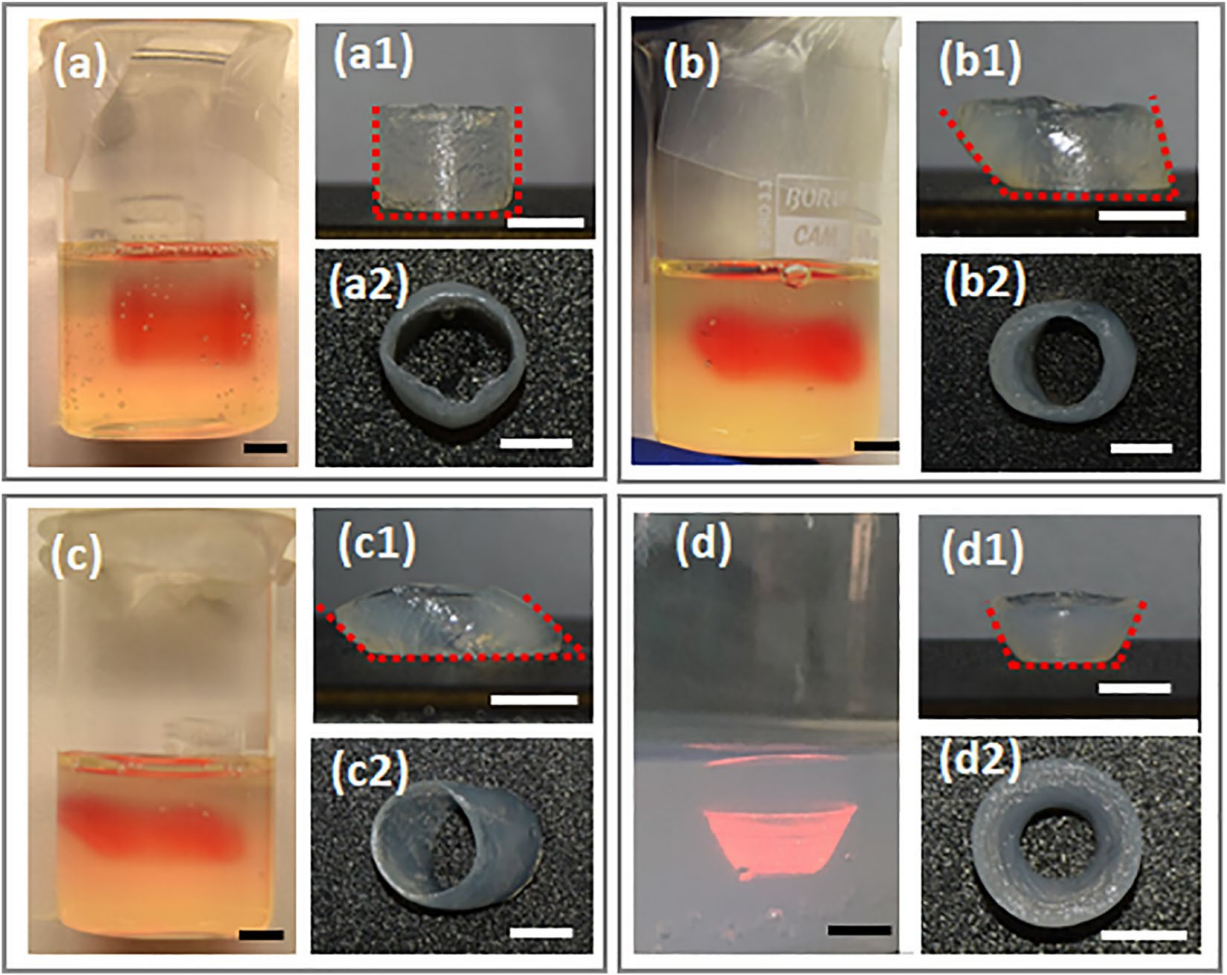
Characterization of PF-RDS support-bath for printability of tubular structures in various angular configurations. Digital images of the printed tubular alginate structures using 25 gauge nozzle in the support-bath angled at (**a**) 90°, (**b**) 60° and (**c**) 45°, and (**d**) a conical structure with 60° angle with respect to the surface. Digital images of front and top views of (a1, a2) 90°, (b1, b2) 60° and (c1, c2) 45° bended tubular structures and (d1, d2) conical structure after removal from support-bath. Scale bars indicate 5 mm.

A detailed overview of 3D printed overhanging hollow structures with different angular configurations before and after recovery from PF-RDS support-bath are demonstrated in Fig. 4. Support-bathes were incubated at 37 °C in a humidified environment for two hours prior printing based on the rheological data explained above. Integrated and bended tubular structures perpendicular to the surface (90°) and with 60° and 45° angles and a conical structure with 60° angle were printed in 20 layers. The front views of the tubular structures in support-bath are presented in Fig. 4a–d. It is clearly seen that the angles of printed structures were the same as designed models. The front and top views of the printed structures after harvesting from support-bath demonstrated that structures were well-crosslinked with integrated layers and preserved angular configurations due to optimized concentration of CaCl_2_ in the PF-RDS bath. The height of the printed structures were measured as 7.58 mm ± 0.9, 4.54 mm ± 0.08, and 3.88 mm ± 0.25 for tubular structures of 90°, 60°, and 45° angles, respectively. Based on computer-aided design (CAD) models, all the printed structures should have the same height. During printing of the tubular structure with 90° angle, the nozzle moved through the same x-y coordinates for 20 times. In contrast, the movement patterns of the nozzle for the other angled structures were not as repetitive in the same coordinates. As a result, diffusion of the hydrogel ink during printing of the structure with 90° angle was more and the final structure had higher height but in a negligible value. Angles of the bended structures after removing from support-bath were measured as follows: 85.9° ± 1.40, 59.4° ± 2.33, and 47.8° ± 5.34, which indicates a high printing resolution for the overhanging CAD models in the PF-RDS bath. The printed structures of alginate with 3% concentration demonstrated that the support-bath had proper viscoelastic characteristics which allowed printing of relatively low viscosity hydrogel in a defined geometry and *in situ* crosslinking while the shape fidelity of overhanging hollow structures were preserved.

**Figure 5:**
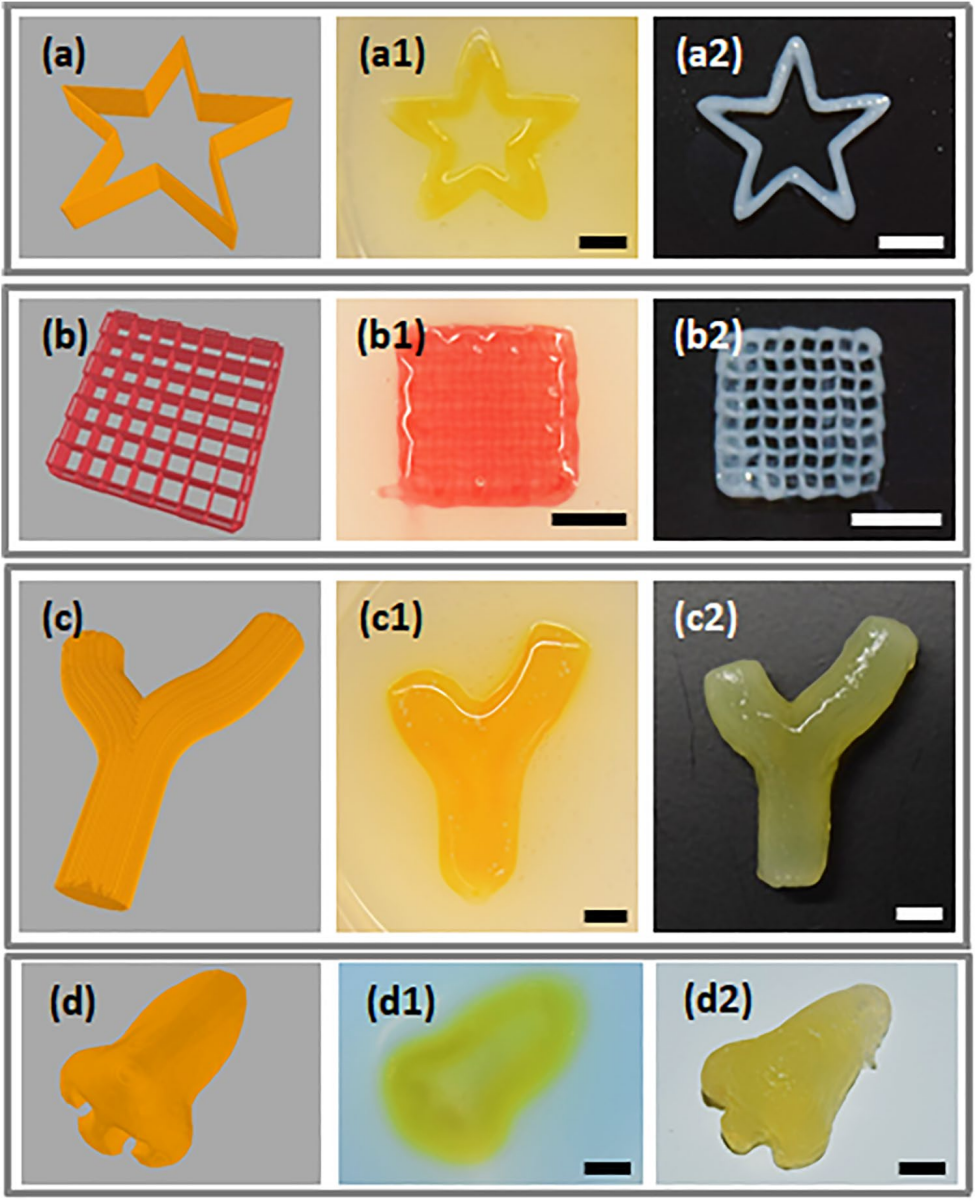
Fabrication of 3D complex constructs. CAD models of (**a**) star shape, (**b**) 0–90° grid pattern, (**c**) branched vascular structure, and (**d**) nose shape. Digital images of the fabricated structures (a1, b1, c1, d1) before and (a2, b2, c2, d2) after recovery from PF-RDS support-bath. Scale bars indicate 5 mm.

Different complex structures including star shape, grid, branched vascular-like and a nose structure were used to demonstrate printing capability of complex geometries with different scales inside the PF-RDS support-bath. Figure 5 shows the CAD models, top views of the printed structures before and after removal of the support-bath. Compared to reported Laponite support bath at which the sample was incubated for 6 h to obtain proper gelation^4^, we could easily remove the printed constructs from the PF-RDS support bath just after printing. Increased concentration of CaCl_2_ in support bath provided enough crosslinking density and ice-cold NaCl solution facilitated the removal of PF coated RDS. A star shape with an outer diameter of 2 cm was selected to demonstrate the precise deposition of extruded filaments with sharp corners (Fig. 5a1). Shape fidelity and its high resolution after the support-bath removal are demonstrated in Fig. 5a2. A small square grid structure with one cm length was chosen to explore the recoverability of the support medium in a repetitive pattern. CAD model of grid structure is depicted in Fig. 5b. Despite the structural integrity and shape fidelity concerns for grid structure printing, the support-bath presented here allowed its fabrication (Fig. 5b1). The structure was harvested without disturbing shape fidelity during removal from the PF-RDS as shown in Fig. 5b2.

A vascular structure plays an important role for living tissues in oxygen and nutrients transportation, and metabolic removal. Fabrication of the vascular network is essential for the functional structures. A CAD model for a branched vascular structure with an overall length of ~3 cm and a width of ~2 cm was designed (Fig. 5c). The vascular structure with a wall thickness of ~0.95 mm and 6 mm height was printed by three offsetted contours in each layer (Supplementary Movie 1). Figures 5c1 shows the printed branched structure inside the support-bath that was printed in 50 min. After gently removal of the support material from the lumen, interconnectivity of the hollow vascular structure was monitored by passing a food dye through in it. The diffusion test demonstrated no leakage from the walls which is illustrated in the supplementary video (Supplementary Movie 2).

To demonstrate the capability of our support-bath for printing of anatomically relevant and 3D complex structures, a nose shape with an overall 2.7 cm length and 1.7 cm width was printed using 25 Gauge nozzle (Fig. 5d). It is to be noted that the printing path strongly affects the final structure as it was demonstrated before^65^. When printing path started from the tip of nose inside the support-bath, we could obtain a smooth surface on the nose with apparent nostrils as represented in Fig. 5d3. This result also verifies the applicability of our support-bath for bioprinting of liquid hydrogels in various complex and large scale structures.

The printed constructs demonstrated the feasibility of the support-bath for continuous and repeated retracing of the print-head. As stress-yielding phenomena happened around the local area where nozzle moves, overall rheological characteristics of the support-bath did not change and did not cause any disruption of the complex shapes, highlighting the stability of the support-bath for long-lasting printing procedures. Although printing speed is considered as a key parameter affecting the yielding properties of the support-bath^14,21^, we printed with different print speeds and the support-bath revealed a consistent recovery. In addition, shape fidelity preservation after structure removal from the support-bath has demonstrated the sufficient integration between the consecutive layers.

### Bioprinting of cell-laden alginate hydrogel in support-bath

**Figure 6:**
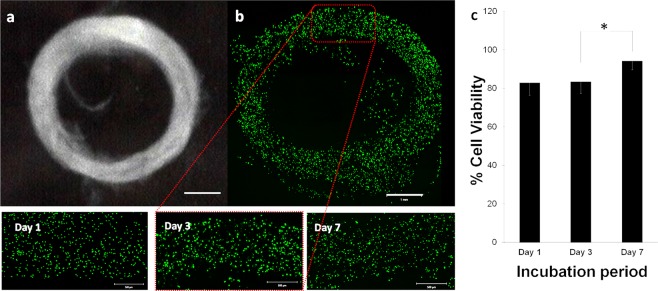
Fabrication of cell-laden alginate constructs using PF-RDS support-bath. (**a**) Image of harvested bioprinted tubular structure from support bath. (**b**) Confocal microscopy image of live/dead cells encapsulated in the alginate hydrogel in a complete 3D bioprinted hollow structure at Day 3 and the zoomed images of cells obtained on Day 1, Day 3 and Day 7. (**c**) Quantitative viability analysis of cells for Day 1, 3 and 7 after bioprinting. Two tail Students *t*-test was used to analyze the significant change in the cell-viability after bioprinting process. P-values *<0.05 were considered as significant. Scale bars indicate 1 mm for (**a**,**b**) and 0.5 mm for the zoomed images.

Cell-laden hydrogels dispensed from a nozzle are being exposed to a shear stress, and followed by an invasive effect of crosslinking mechanism^61,66^. Therefore, fabrication process of cell-laden hydrogels inside the support-bath might affect the cellular integrity. Since the nozzle size and feeding pressure for the extrusion of cell-laden hydrogel have an inverse relation, the use of small needle size might have more negatively effect on cell viability. We selected a small nozzle (30 Gauge) to investigate the effect of bioprinting process in support-bath on cellular integrity in intense conditions. Compatibility of the bioprinting process was evaluated by monitoring cell viability after bioprinting. Alginate used to encapsulate the cells in this study is commonly employed in 3D bioprinting applications due to the biocompatibility and fast crosslinking in the presence of Ca^2+^ ions despite its bio-inert nature and limited biodegradability. It is also used as a thickening hydrogel to enhance the bioprintability of the other, more bioactive hydrogels^4,14^. NIH-3T3 mouse fibroblast cells in a density of 1 × 10^6^ cells/mL were encapsulated in 3% of alginate and bioprinted by feeding pressure of 0.5 bar and print speed of 150 mm/min. Well defined 3D hollow structure with a 5 mm diameter and average height of 0.6 mm was obtained by the integration of deposited four concentric fibers of cell-laden alginate. Figure 6 shows (a) bright field image of harvested bioprinted structure from the support-bath and (b) Calcein AM (green) and PI (red) stained, live and dead cells, respectively, in a complete bioprinted structure. The results demonstrated the structural integrity of the proposed structure for bioprinting and their efficient recovery from support-bath with high percentage of viable cells^4,14,22^. Live and dead cell numbers showed that 82.7 ± 6.5% cells in alginate hydrogel were viable after one day (Day 1) of incubation (Fig. 6c). The percentage of viable cells did not change on Day 3 while the cell viability increased to 94.3 ± 4.6% at Day 7. The results indicated that extrusion pressure did not affect the cell viability significantly and the cells almost recovered at Day 7.

These results demonstrate that the bioprinting process in PF-RDS support-bath does not cause significant damage to the cells encapsulated within the hydrogel. Further incubations allow cell recovery as observed on viability results. The developed support-bath bioprinting process is not limited to alginate and can be used for other hydrogels. The long-term cell viability activity evaluations are necessary for bioactivity investigation of different hydrogels^61,66^.

## Conclusion

We herein developed a composite gel based on PF-RDS as a sacrificial support material for in-gel printing of overhanging complex and hollow structures. Alginate was used as the printing bioink and CaCl_2_ was utilized to enhance the viscoelastic properties of the polymer-nanoclay composite gel and as the crosslinking agent for alginate. The effects of different concentrations of PF and CaCl_2_ on rheological properties including viscoelasticity, yield, and recoverability showed the potential of our composite structure to be utilized as a supporting material with the ability of long-term storage and reusability. Utilization of PF inside the support-bath resulted in increased tolerance of nanoclay discs over the CaCl_2_ content and allowed to dispense less viscous hydrogel with higher shape fidelity at low extrusion pressure, which would be a more cell friendly process. Furthermore, it decreased the time of incubation period in the support-bath that provided enough crosslinking density before removing the fabricated construct. Compared to the previously published studies which used only Laponite for their support-bath^4,14^, the printed structures could be harvested easier without significant residues of the support-bath adhered to the structures. Performance of the composite gel as a support bath was validated by printing several alginate hydrogel structures. The measured dimension of the recovered hollow structures showed that the height and the angles were well defined. The support-bath did not only allow precise printing of different complex structures but also helped recovery of the structures with high resolution. A diffusion test assessed on a printed branched vascular structure also demonstrated structural integrity and interconnectivity of the channels. NIH-3T3 cell-laden alginate bioprinted inside the support-bath showed homogeneous distribution with above 80% viability within the hydrogel revealed the potential of the composite support structure for a cell friendly bioprinting process and to be further used for various tissue engineering applications.

## Methods

### Preparation of PF-RDS support-bath and characterization

PF-RDS support-bath was prepared by slowly adding equal volume of PF-CaCl_2_ (Sigma Aldrich) solution into Laponite-RDS (BYK Additives & Instruments) suspension. Briefly, PF solutions with 15%, 20% and 25% concentrations were prepared by dissolving in 0.5, 1 or 2% (w/v) CaCl_2_ solution at cold room (4 °C) under continuous stirring. A 6% Laponite-RDS solution was prepared by suspending appropriate amount of dry Laponite-RDS powder in deionized (DI) water and vigorously stirring for minimum one hour at room temperature to allow fully exfoliation and dispersion of nanoclay particles with a transparent appearance^4,54^. Then, PF solution was added slowly into Laponite-RDS solution under continuous stirring at 4 °C to obtain final concentrations of 7.5, 10 and 12.5% for PF and 0.25, 0.5 and 1% of CaCl_2_ with constant Laponite concentration of 3%. The mixture was further stirred for minimum one hour at 4 °C. The composite material was centrifuged at 4000 rpm for 3 min prior to incubation at 37 °C to remove the bubbles. It is worth to mention that final 2% of Laponite was also prepared and examined but electrical repulsive forces were not enough to make an ordered array of particles. Hence, the matrix storage and recovery were not appropriate for printing^33,46,48^ and it was omitted from further experiments (data not shown). The composite material was stable and could be stored at 4 °C for a long time with no changes in its properties^49^. Based on rheological data, 3% of Laponite-RDS, 10% of PF, and 0.5% of CaCl_2_ concentrations were selected for the support bath formulation.

### Rheological measurements

All rheological characterizations were performed on a MCR302 (Anton Paar, Austria) equipped with a Peltier plate for temperature control. A stainless steel parallel plate of 25 mm diameter with a gap distance of 0.5 mm was utilized for all the experiments. A low viscosity silicon oil was used as the solvent trap during the measurements. Rotational and oscillatory measurements were performed to investigate the flow and viscoelastic behavior of the support-baths. Gel yield stresses and linear viscoelastic (LVE) regions were measured by strain sweep from 0.01–100% at a constant angular frequency of 10 rad/s. Oscillatory angular frequency sweeps were carried out within the LVE range (0.6% strain and angular frequency of 0.1–100 rad/s) to monitor the dynamic rheological behavior. Temperature sweep experiments were conducted from 4–37 °C with 5 °C/min ramp to observe gelation temperature and evolution of the structure’s moduli. To investigate the recovery behavior of the support-bath during hydrogel extrusion, cyclic strain test at low and high oscillatory strains of 0.6 and 50% at constant angular frequency of 10 rad/s and 10 s duration per cycle was performed. Shear rate sweeps were conducted to monitor the shear thinning behavior and viscosity changes of the formulations between 0.01–100 1/s.

Samples were incubated at 37 °C for 2 h with a pre-shear rate of 1/s prior to each run. Three measurements were taken for each sample and mean values were reported. The effect of different compositions of PF and CaCl_2_ on flow behavior of the support material was investigated. Control groups were selected as CaCl_2_-PF named as Control 1 and CaCl_2_-Laponite RDS named as Control 2 with different concentrations of CaCl_2_. Control 1 contained 10 and 1% for PF and CaCl_2_, respectively to observe the effect of Laponite on rheological properties. Control 2 were included constant Laponite-RDS of 3% and 0.125, 0.25, 0.5, and 1% of CaCl_2_.

### CAD modelling of complex structures and 3D printing inside support-bath

A customized three axes 3D bioprinter controlled by MACH3 software (Newfangled Solutions) was used to print different structures. Hydrogel solution was loaded into a 10 mL material reservoir equipped with a double thread screwed plastic nozzle (Musashi Engineering, Japan) and material extrusion from the printing head was provided through a pneumatic dispensing unit (Nordson EFD Performus V). CAD models of the constructs were developed in Rhinoceros 6 (Robert McNeel &Associates, USA) and tool paths were generated and transformed into G-codes.

Star shape, grid pattern, branched vascular-like, and a nose shape structure in different scales were printed using 3% (w/v) sodium alginate (Sigma Aldrich) solution prepared in DI water. Star and grid-pattern structures were printed with a 25 Gauge nozzle and vascular-like and nose shape were fabricated via a 23 Gauge nozzle. For star shape printing, 15 layers of hydrogels were deposited by setting parameters as 140 mm/min print speed and 0.7 bar feeding pressure. The structure was formed by depositing two concentric contours without any gap in between them. The grid shape of 0–90° zig-zag 10 × 10 mm^2^ deposition pattern including 9 stripes with 750 µm gap in between was extruded in each layer with 140 mm/min print speed and 0.7 bar feeding pressure. Branched vascular-like construct was printed at 150 mm/min print speed and 0.6 bar feeding pressure. Nose shape structure was printed with 2.7 cm length and 1.7 cm width using a 25 Gauge nozzle at 130 mm/min print speed and 0.5 bar feeding pressure by three offsetted counters.

Prior to support-bath removal, the structures were post-crosslinked in a 2% CaCl_2_ solution for 10 min. Then, residual support-bath materials were removed from the beakers by pipetting 1% ice-cold NaCl solution. To show the presence of lumen inside the structure and impermeability and its interconnectivity, diffusion test was performed by delivering blue food dye solution from one end of the branched vascular structure (Supp. Video 1).

### Bioprinting of cell-laden alginate in PF-RDS support-bath

All components of support-bath and alginate were sterilized by autoclaving. Dry powder of PF was sterilized at 105 °C for 30 min as suggested in the previous study to prevent rheological property changes^67^. CaCl_2_ and Laponite-RDS were also autoclaved in their powder forms. Then, the support-bath was prepared as explained above. Alginate solution was prepared in 3% (w/v) concentration in 1 × PBS and autoclaved at 121 °C for 15 min before encapsulating the cells.

NIH-3T3 cells (ATCC) were grown in Dulbecco’s Modified Eagle Medium (DMEM, Sigma) supplemented with 10% fetal bovine serum (FBS, Sigma) and 1% penicillin-streptomycin (Gibco) at humidified atmosphere containing 5% CO_2_ at 37 °C. The cells with a 1 × 10^6^ cells/mL density were prepared by suspension in 3% alginate solution at room temperature. A 0.5 bar pressure was applied to extrude cell-laden hydrogel from a 30 Gauge nozzle with 150 mm/min print speed. After printing, the alginate structures were washed with 1% ice-cold NaCl solution and DMEM. They were placed into 12 well-plate with fresh DMEM and incubated at the incubator.

### Evaluation of in-gel bioprinting biocompatibility

The viability of 3T3 cells encapsulated in the alginate was evaluated on Day 1, Day 3, and Day 7 after bioprinting. At the end of incubation points, the samples were transferred into glass bottom Petri dishes and washed with 1 × PBS. Calcein AM/PI staining was used to evaluate live/dead cells. Briefly, cells were first stained with 1 μM calcein-AM (Invitrogen, green fluorescence) for 30 min and then, with 0.75 μM propidium iodide (Invitrogen, red fluorescence) for 5 min in 1 × PBS at 37 °C, followed by washing in 1  × PBS for three times. The viable cells were monitored with maximum excitation/emission wavelengths of 488/515 nm, respectively while dead cells were monitored at maximum excitation/emission wavelengths of 561/625 nm, respectively, using inverted confocal microscope (Carl Zeiss LSM 710). 3D images were obtained using tiled z stacks with 5.00 µm intervals in 2.77 µm pixel size. Live/dead cells were analyzed quantitatively by using ImageJ 1.48 v software.

### Statistical analysis

All values for cell viability and rheological assessments are presented as the mean ± SD (n=3). P Students *t*-test was used to analyze the significant difference. P values <0.05 and <0.01 are considered statistically significant.

## Supplementary information

Supplementary Movie 1.

Supplementary Movie 2.
